# Antibiotic Use in Communities

**DOI:** 10.3390/antibiotics13050438

**Published:** 2024-05-13

**Authors:** Timo J. Lajunen

**Affiliations:** 1 Department of Psychology, Norwegian University of Science and Technology, NO-7491 Trondheim, Norway; timo.lajunen@ntnu.no; 2 Department of Social Sciences, School of Humanities and Social Sciences, University of Nicosia, CY-1700 Nicosia, Cyprus

## 1. Introduction

Since their discovery and clinical introduction in the 1930s–1940s, penicillin and sulphonamides have had a profound impact on public health [1]. Antibiotics have become the most frequently prescribed medications worldwide, with their usage steadily increasing. From 2000 to 2015, there was a notable 65% surge in antibiotic consumption, as measured by defined daily doses, and a 39% increase in the antibiotic consumption rate per 1000 individuals per day [2]. This rapid escalation in antibiotic use was predominantly observed in low- and middle-income countries [2]. Contributing factors include the availability of more affordable generic antibiotics, rising incomes, and a lack of regulatory measures, all of which are likely to fuel the consumption of antibiotics further.

Simultaneously, the heightened utilisation of antibiotics in humans, animals, and agriculture has given rise to a significant global health concern: antimicrobial resistance. This phenomenon has rendered the treatment of numerous prevalent infectious diseases more challenging and has resulted in an escalation of adverse effects, disability, and mortality. It has been estimated that bacterial antimicrobial resistance directly caused 1.27 million deaths worldwide in 2019 and contributed to an additional 4.95 million deaths [3]. Antimicrobial resistance significantly complicates treatment, necessitating additional diagnostic investigations, therapies, prolonged hospital stays, and ultimately, augmented healthcare expenditures. The World Health Organisation has issued a warning that failure to take immediate action may result in the advent of a post-antibiotic era, wherein commonplace infections and minor injuries could once again prove fatal [3].

Considerable variations in the utilisation of antibiotics can also be observed across different healthcare sectors. In Europe, for example, there is a significant discrepancy in antibiotic consumption between the human outpatient and hospital sectors, with the former exhibiting a tenfold higher usage rate [4]. In its “People-centred approach to addressing antimicrobial resistance in human health”, the WHO highlights the importance of community and civil society empowerment in antibiotic resistance [5]. Resource allocation to enhance community awareness and education and foster behavioural changes is necessary to combat antimicrobial resistance [5]. The development of community-driven solutions should be rooted in a more comprehensive comprehension this pressing issue [5].

## 2. Overview of the Published Articles

This Special Issue, “Feature Papers in Antibiotic Use in the Communities”, presents eight studies about community antibiotic use and stewardship.

Statistics show a vast geographical variation in community antibiotic use, even within European countries [4]. The studies included in this is Special Issue represent an impressive sample of nationalities and countries, including Americans [6], Cypriots (Greek and Turkish) [7], Dutch [8], Germans [9], Greeks [10], Italians [11], Syrians [12], Thai [13], and Turks [10]. Since antibiotic use and, consequently, antimicrobial resistance vary significantly between cultures and communities, it is essential to investigate the reasons behind excessive antibiotic use in as many countries and cultures as possible. In some cultures and communities, antibiotic use, especially self-medication with leftover antibiotics, may reflect a lack of knowledge, with antibiotics being used as a general medication for viral infections such as the common cold and sore throat. In an earlier study conducted in Turkey, 51.4% thought that antibiotics are effective in treating viral diseases, and 34.2% had self-medicated themselves with antibiotics [14]. Antibiotic overuse might also reflect general worry and health-related anxiety; antibiotics are used as a precaution [14]. In addition, patients may expect and pressure physicians to prescribe antibiotics. Lajunen et al. [10] and Sullman et al. [7] investigated whether respondents had requested antibiotics from their physicians. Their findings indicate that 11.0% of respondents in Greece, 10.8% in Turkey, 18.8% in South Cyprus, and 6.8% in North Cyprus reported asking for antibiotic prescriptions [7,10]. Such pressure could lead physicians to adopt a more lenient policy on prescribing antibiotics in order to meet patient expectations. It is also possible that excessive antibiotic use reflects the high cost and unavailability of medical care in some communities. When medical care is expensive or difficult to reach, antibiotics might be taken just in case the infection is bacterial. To enhance community awareness about antibiotics and behavioural change, it is essential to know the local community-specific risk factors related to excessive antibiotic use in the first place. This Special Issue, with studies from a number of countries, increases our knowledge about the risk factors behind antibiotic use in communities.

Extensive uses of antibiotics can be observed in medicine, dentistry, veterinary practice, and agriculture [15]. Therefore, antibiotic misuse and antibiotic resistance should be seen holistically as one topic encompassing these different fields and should be studied using various research approaches, methods, and data sources. Seven of the studies included in the present Special Issue investigated antibiotic use in medicine, while one study analysed data on antibiotic prescriptions in primary dental care in Germany from 2017 to 2021 [9]. Six of the studies employed a cross-sectional survey about antibiotic use, attitudes to and knowledge about antibiotics, and antimicrobial resistance [6,7,10,11,12,13], while one study collected data with qualitative focus group interviews [8]. All these distinctive methods have the potential to reveal different aspects of antibiotic use.

Gradl and colleagues [9] analysed antibiotic prescription patterns in primary dental care in Germany from 2017 to 2021. They discovered that amoxicillin was the predominant antibiotic prescribed, followed by clindamycin. Notably, dental prescriptions constituted 56% of all clindamycin prescriptions in primary care in 2021 [9]. This indicates that while there has been some improvement in the overuse of clindamycin in German dentistry, the issue still persists [9]. 

Değer et al. [12] and Lescure et al. [8] investigated antibiotic use among minorities. Değer et al.’s sample consisted of 542 Syrian immigrants in Turkey [12], while the qualitative, focus-group-based study conducted by Lescure included Syrian immigrants and immigrants from Cape Verde, Morocco, Surinam, and Turkey [8]. Among Syrian immigrants, female gender, older age, lower education level, and regular medication use were related to lower health literacy [12]. In this sample, 80.3% had limited health literacy, indicating the necessity for specific interventions to improve health and facilitate societal integration [12]. In the study by Lescure et al. [8], no significant differences were observed between immigrant and native Dutch participants regarding their attitudes towards antibiotics. Notably, differences within each group were more pronounced than differences between the groups [8]. Participants displayed varied behaviours, including adopting assertive attitudes to obtain antibiotics, possessing limited knowledge about antibiotics, or utilising antibiotics incorrectly. Both studies emphasise the importance of communicating the correct use of antibiotics regardless of the patient’s background. 

Lajunen et al. [10], Niyomyart et al. [13], Peinnino et al. [11], and Sullman et al. [7] studied antibiotic use and knowledge using a structured cross-sectional survey among the majority population. These studies show that knowledge about antibiotic effectiveness varies among countries but is generally rather low. In a Thai sample, only 31.7% of respondents knew antibiotics are ineffective against the common cold and the flu [13]. In comparison, 73.9% of Greek and 63.7% of Turkish respondents knew that antibiotics are ineffective in treating the common cold [10]. Among Greek and Turkish Cypriots, these figures were 41.9% and 75.0%, respectively [7]. In Italy, only 46.20% knew that antibiotics are ineffective against viruses [11]. These studies investigated various factors related to the misuse of antibiotics. A general finding seems to be that background factors like education level and having children are related to antibiotic use [11,13], and that knowledge level and attitudes are strong predictors of using antibiotics correctly [7,10,11,13]. Obviously, educational campaigns about correct antibiotic use and the risks of antibiotic resistance are needed. These campaigns should be tailored according to the target community.

McCracken et al.’s [6] survey combined structured forced-choice questions with open-ended questions about antibiotic resistance and subjected the responses to qualitative analysis. The answers about antibiotic resistance were coded into the following themes: 35% bacteria adaptation, 22% misuse/overuse, 22% resistant bacteria, 10% antibiotic ineffectiveness, 7% body immunity, and 3% incorrect definition [6]. A statistically significant difference in responses was found between those who reported having shared an antibiotic versus those who had not. The authors concluded that future campaigns should focus on improving public awareness of antibiotic resistance and modifying behaviours that can contribute to resistance [6].

## 3. Conclusions

These eight studies, conducted in diverse countries and addressing various aspects of antibiotic use and resistance, consistently reveal a common finding. There is considerable variation in knowledge of antibiotics and attitudes towards their use across different cultures and communities. Overall, there is a low level of awareness regarding the types and functions of antibiotics and antibiotic resistance. This lack of understanding is often linked to the misuse of antibiotics, including practices such as self-medication, storing antibiotics for future use, and sharing antibiotics. A lack of awareness among patients can result in heightened expectations for physicians to prescribe antibiotics with minimal justification. These expectations may pressure physicians to prescribe antibiotics ‘just in case’ in order to satisfy patient demands. These findings underscore the crucial need to dispel misconceptions about antibiotics. Targeted campaigns and interventions aimed at improving knowledge and promoting responsible antibiotic use should be tailored to suit the specific needs of each community.
